# Development and initial evaluation of a clinical prediction model for risk of treatment resistance in first-episode psychosis: Schizophrenia Prediction of Resistance to Treatment (SPIRIT)

**DOI:** 10.1192/bjp.2024.101

**Published:** 2024-09

**Authors:** Saeed Farooq, Miriam Hattle, Tom Kingstone, Olesya Ajnakina, Paola Dazzan, Arsime Demjaha, Robin M. Murray, Marta Di Forti, Peter B. Jones, Gillian A. Doody, David Shiers, Gabrielle Andrews, Abbie Milner, Maria Antonietta Nettis, Andrew J. Lawrence, Danielle A. van der Windt, Richard D. Riley

**Affiliations:** School of Medicine, Keele University, Newcastle-under-Lyme, UK; National Institute for Health and Care Research (NIHR), UK; and St George's Hospital, Midlands Partnership University NHS Foundation Trust, Stafford, UK; Institute of Applied Health Research, College of Medical and Dental Sciences, University of Birmingham, Birmingham, UK; and National Institute for Health and Care Research (NIHR) Birmingham Biomedical Research Centre, Birmingham, UK; Department of Biostatistics & Health Informatics, Institute of Psychiatry, Psychology and Neuroscience, King's College London, London, UK; and Department of Behavioural Science and Health, Institute of Epidemiology and Health Care, University College London, London, UK; Department of Psychological Medicine, Institute of Psychiatry, Psychology and Neuroscience, King's College London, London, UK; Department of Psychosis Studies, Institute of Psychiatry, Psychology and Neuroscience, King's College London, London, UK; Department of Psychosis Studies, Institute of Psychiatry, Psychology and Neuroscience, King's College London, London, UK; and Department of Psychiatry, Experimental Biomedicine and Clinical Neuroscience, University of Palermo, Palermo, Italy; Social, Genetic and Developmental Psychiatry Centre, Institute of Psychiatry, Psychology and Neuroscience, King's College London, London, UK; Department of Psychiatry, University of Cambridge, Cambridge, UK; Division of Psychiatry and Applied Psychology, University of Nottingham, Nottingham, UK; St George's Hospital, Midlands Partnership University NHS Foundation Trust, Stafford, UK; Institute of Applied Health Research, College of Medical and Dental Sciences, University of Birmingham, Birmingham, UK; and National Institute for Health and Care Research (NIHR) Birmingham Biomedical Research Centre, Birmingham, UK.; School of Medicine, Keele University, Newcastle-under-Lyme, UK; Psychosis Research Unit, Greater Manchester Mental Health NHS Trust, Manchester, UK; and University of Manchester, Manchester, UK; South London and Maudsley NHS Foundation Trust, London, UK; and Department of Psychosis Studies, Institute of Psychiatry, Psychology and Neuroscience, King's College London, London, UK

**Keywords:** First-episode schizophrenia, treatment resistant, prognostic model, decision analysis, mixed methods

## Abstract

**Background:**

A clinical tool to estimate the risk of treatment-resistant schizophrenia (TRS) in people with first-episode psychosis (FEP) would inform early detection of TRS and overcome the delay of up to 5 years in starting TRS medication.

**Aims:**

To develop and evaluate a model that could predict the risk of TRS in routine clinical practice.

**Method:**

We used data from two UK-based FEP cohorts (GAP and AESOP-10) to develop and internally validate a prognostic model that supports identification of patients at high-risk of TRS soon after FEP diagnosis. Using sociodemographic and clinical predictors, a model for predicting risk of TRS was developed based on penalised logistic regression, with missing data handled using multiple imputation. Internal validation was undertaken via bootstrapping, obtaining optimism-adjusted estimates of the model's performance. Interviews and focus groups with clinicians were conducted to establish clinically relevant risk thresholds and understand the acceptability and perceived utility of the model.

**Results:**

We included seven factors in the prediction model that are predominantly assessed in clinical practice in patients with FEP. The model predicted treatment resistance among the 1081 patients with reasonable accuracy; the model's C-statistic was 0.727 (95% CI 0.723–0.732) prior to shrinkage and 0.687 after adjustment for optimism. Calibration was good (expected/observed ratio: 0.999; calibration-in-the-large: 0.000584) after adjustment for optimism.

**Conclusions:**

We developed and internally validated a prediction model with reasonably good predictive metrics. Clinicians, patients and carers were involved in the development process. External validation of the tool is needed followed by co-design methodology to support implementation in early intervention services.

Treatment-resistant schizophrenia (TRS) is defined as the presence of persistent symptoms of at least moderate severity and functional impairment despite treatment with at least two different antipsychotics used in adequate dose, each for 4–6 weeks’ minimum duration.^1^ Results from a recent meta-analysis showed that almost a quarter of people with first-episode psychosis (FEP) or schizophrenia develop TRS in the early stages of treatment, increasing to one-third when the estimates included those who relapse despite initial response and long-term follow-up.^2^ In England, 25–50% of the National Health Service's (NHS) £11.8 billion mental health budget is allocated to schizophrenia care.^3^ Considering that about one-third of people with schizophrenia develop TRS, treatment resistance accounts for a large proportion of these costs.

## Current interventions

Clozapine is the only medication licensed for TRS, and is often delayed by between 5 and 10 years.^4,5^ Other interventions that can be used in TRS, such as cognitive–behavioural therapy (CBT), are used even less optimally.^6^ The delay in starting effective treatments for TRS is due to multiple factors, including a lack of clear guidance on the identification of TRS and the difficulties in identifying suitable patients.^7^ A systematic review demonstrated that delayed clozapine use was related to poor treatment outcomes among people with TRS.^8^ Another recent systematic review based on 34 articles involving 9386 people with schizophrenia spectrum disorders suggested that starting clozapine early in treatment could indeed improve the outcome of people with first-episode schizophrenia.^9^ Specifically, Yoshimura et al (2017) showed that when delay in initiating clozapine was less than 2.8 years, the response rates were 81.6%, compared with 30.8% when clozapine was delayed by 2.8 years or more.^10^

The early identification of those at risk of treatment resistance would minimise the delays to effective treatments for TRS, thus preventing much greater disability, frequent hospital admissions and other complications associated with TRS.^11^ A systematic review of prediction models in FEP^12^ identified only two studies describing the prediction of TRS.^13,14^ These studies had several methodological limitations, including small sample size and lack of adequate power for robustly developing a prediction model.^12^

## Our model

To address this, we combined data from two cohorts to develop a prognostic model for estimating an individual's 5- to 10-year risk of developing treatment resistance to standard (non-clozapine) antipsychotic drugs based on sociodemographic and clinical characteristics, and to undertake internal validation of the model's predictive performance.

## Method

A protocol following the PROGRESS (prognosis research strategy) framework and guidance for prognosis research describing full methodology has been previously published.^15^ We describe the key methodological details here. We used the TRIPOD (transparent reporting of a multivariable prediction model for individual prognosis or diagnosis) guidelines to report the study (see Supplementary Appendix 1, available at https://doi.org/10.1192/bjp.2024.101).^16^ We used a mixed-methods design including statistical methods to develop the prognostic model using individual participant data from two existing cohorts and examined its predictive performance, followed by qualitative data generated through focus groups and semi-structured interviews with clinicians (psychiatrists, pharmacists) responsible for managing people with TRS to explore the acceptability and perceived utility of the prediction model.^15^

### Study sample

Individual participant data from two existing cohorts (GAP and AESOP-10) that included people with FEP presenting to mental health services were used to develop and internally validate the prognostic model.

#### The Genetics and Psychosis (GAP) cohort

The Genetics and Psychosis (GAP) cohort comprised participants aged 18–65 years and meeting criteria for FEP (ICD-10 diagnoses: F20.0, F25.0, F28.0, F29.0),^17^ validated by administration of the Schedules for Clinical Assessment in Neuropsychiatry (SCAN).^18^ GAP inclusion and exclusion criteria are described elsewhere.^19^ Approximately 5 years after first contact with mental health services for psychosis in South East London, all patients with a baseline diagnosis of schizophrenia spectrum disorders and who had given consent for follow-up and assessment of their clinical records, were followed up. Follow-up data, including treatment received for psychosis, were extracted retrospectively using the electronic psychiatric clinical records (EPCRs). All deaths and emigrations up to and including those that occurred during the final year of follow-up were identified by a case-tracing procedure with the Office for National Statistics (ONS) for England and Wales and the General Register Office (GRO) for Scotland. Overall, 246 out of 283 participants were successfully followed up (86.9%); sufficient information on treatment received was available for 239 individuals. Eighty (33.5%) of these individuals met criteria for treatment resistance and 159 (66.5%) were non-treatment resistant. Patients who were lost to follow-up were not different in the baseline characteristics from patients who had full follow-up data (Supplementary Table 3 in Ajnakina et al (2017)^20^).

#### The Aetiology and Ethnicity in Schizophrenia and Other Psychoses (AESOP-10) cohort

Aetiology and Ethnicity in Schizophrenia and Other Psychoses (AESOP-10) is a longitudinal, population-based study of incident cases of psychosis from defined catchment areas with a 10-year follow-up.^14^ At baseline, all patients aged 16–64 years who presented with a first episode of a functional psychosis over a 2-year period in services in South East London and Nottingham (UK) were invited to take part. Psychopathology was assessed using the SCAN.^18^ Diagnoses were made according to the ICD-10 diagnostic criteria for research during clinical consensus meetings. Out of a total of 557 participants recruited at baseline, 434 provided follow-up data (77.9%). For 286 participants for whom there was complete information on medication, adherence to treatment and symptom ratings over the 10-year follow-up period were recorded in the individual participant data. Of the 434 participants, 88 (20.3%) met the criteria for TRS. The comparison of those who had complete information at follow-up and those without revealed that there was no evidence of systematic differences by age, gender, duration of untreated psychosis and other sociodemographic and clinical variables (Supplementary Table 3 in Morgan et al (2014)^21^).

### Baseline data

Baseline data were collected within a 3-month period following the first contact with psychiatric services to ensure minimal effect of exposure to antipsychotic medications. The following variables were recorded at baseline in both data-sets: age at first contact with mental health services, gender, ethnicity, IQ, educational level, employment status, living arrangements, living alone, being single or separated, baseline diagnosis (ICD-10 and DSM-IV), mode of onset, duration of untreated psychosis, family history of psychosis, substance misuse, alcohol use, symptom dimensions and childhood adversity.

### Outcome measure

The primary outcome measure was the occurrence of TRS at 5 to 10 years. As the follow-up was 5 years in GAP and 10 years in AESOP-10, and outcome status was unknown by 5 years (AESOP-10) or 10 years (GAP), to utilise both data-sets for model development we assumed that risks by 5 and 10 years were similar. Based on participants with complete outcome data, the overall outcome risk was 0.263 across both data-sets combined, 0.267 in GAP (5-year follow-up) and 0.259 in AESOP (10-year follow-up), indicating that the data-sets and time-points were very similar in terms of the overall risk for TRS.

#### Treatment resistance definition

In keeping with the consensus definition of TRS,^1^ treatment resistance in both data sources was defined as not responding to two consecutive periods of antipsychotic medication of adequate dose and for an adequate duration and/or the documented reason for switching antipsychotic medication being a lack of therapeutic response.^13,14,22–24^ The adequate daily dose of antipsychotics was defined as equivalent to a daily dose of ≥400 mg chlorpromazine.^25^ This definition of TRS requires treatment with two antipsychotics used in therapeutic dose in a sequential manner, which is rare in clinical practice. A gap of no longer than 14 days was allowed between consecutive treatments. We only considered as meeting criteria for TRS those individuals who failed to respond and not those who were intolerant of antipsychotic medications or those who themselves discontinued antipsychotic medication.^13,14,22–24^ A lack of improvement in symptoms and social functioning was defined as the continued presence of overt psychotic symptoms (operationalised^13^ as a score of 2 or 3 on rating scale 2 in the SCAN, where 0 = absence, 1 = symptom occurred, but fleeting, 2 = symptom definitely present, 3 = symptom present more or less continuously) for 6 months or longer.^21,26,27^

### Characteristics of the data-sets

The salient features of the data-sets and cohorts are given in Table 1.

**Table 1:** Salient features of the data-sets and cohort

	AESOP-10	GAP
Calendar years of recruitment	1997–1999	2005–2010
Catchment areas	South East London and Nottingham	South East London
Follow-up period	10 years	5 years
Participants at baseline, *n*	557	283
Participants with data at follow-up, *n*	434	246
Patients meeting criteria for treatment-resistant schizophrenia	88 (20.3%)	80 (33.5%)

GAP, Genetics and Psychosis cohort; AESOP-10, Aetiology and Ethnicity in Schizophrenia and Other Psychoses.

### Identification of candidate predictors

We used an iterative approach to identify candidate predictors for model inclusion *a priori* (before data analysis) (Supplementary Appendix 2). This included:

- a review of relevant literature,^20,28–33^ with additional focused searches (MEDLINE, Embase, PsycInfo)
- acknowledgement of which variables were recorded in both data-sets (see above)
- consultation with our expert and patient advisory groups, through face-to-face (patient advisory group) and videoconference (clinical expert advisory group) meetings. These meetings were facilitated by an experienced qualitative researcher (T.K.) and clinical member of the study team (S.F.).

We identified 22 candidate predictors from a literature review using the above approach and finally selected the following seven predictors for model development, as these were available in both data-sets and were ranked as most important by the expert panel in predicting TRS:

- age^34^
- duration of untreated psychosis^20^
- comorbidity: drug use (yes/no)^35^
- alcohol use (yes/no)^21,36^
- premorbid adjustment (National Adult Reading Test; NART)^37^
- diagnosis of schizophrenia or schizoaffective disorder (instead of psychotic depression or psychotic mania)^19^
- family history of psychosis.^38^

The details of the definition and measurement of these candidate predictors are provided in Supplementary Appendix 7.

### Qualitative data collection

A topic guide and real life case examples (with fictitious names and identifying information) (Supplementary Appendix 5) were used to prompt discussion of challenges and opportunities for designing and embedding a clinical decision tool for TRS, based on the prediction model. The topic guide was developed with input from the patient advisory group. All data collection took place online via Microsoft Teams.

### Statistical analysis for model development

#### Handling of missing data

We examined the extent and distribution of missing data for predictors, treatment characteristics and clinical outcomes and compared these with data for those who were lost to follow-up to assess the risk of attrition bias. Imputation of missing values was handled separately for each cohort, prior to combining the data for predictive modelling, to retain any potential between-cohort heterogeneity. Imputation of missing data (both outcomes and predictors) was done using multiple imputation of 50 data-sets, under a ‘missing at random’ assumption, including outcome and candidate predictors in the imputation model alongside a broader set of auxiliary variables available in each cohort (to improve the missing at random assumption).^39,40^ The complete methods of imputation are provided in Supplementary Appendix 10.

### Sample size for model development

There was a large quantity of missing data across both data-sets for the outcome of treatment resistance, and therefore two sample-size calculations were performed, one based on cases with complete outcome data (*n* = 638) and one based on all available cases (*n* = 1081). Of the 638 participants with complete outcome data, 168 were treatment resistant, corresponding to an outcome event prevalence of 0.263. Based on this sample size and outcome proportion, and conservatively assuming a Nagelkerke *R*^2^ of 15%, the minimum sample size calculation of Riley et al^41^ indicated that a maximum of 8 predictor parameters could be considered. When including all participants with missing outcome data and assuming the same prevalence and Nagelkerke *R*^2^, the sample size calculation suggested that a maximum of 13 predictor parameters could be considered. Although imputation was used to allow inclusion of patients with missing outcome data this does not recover all information. Therefore, we allowed for a maximum of 10 predictor parameters in the model, a compromise between the 8 and 13 from the two calculations.

#### Non-linear transformations of predictors

Non-linear transformations for continuous predictors were investigated using fractional polynomials and for missing observations imputed using multiple imputation of covariates by fully conditional specification using the substantive model. There was no evidence for non-linear transformations for the continuous predictors in the model. Therefore, all continuous covariates were modelled as linear.

#### Model development and internal validation

A model for predicting risk of TRS was developed based on penalised multivariable logistic regression, considering all candidate predictors emerging from the process as described above. A logistic model (with treatment resistance at 5–10 years as the outcome) with a random intercept for each data-set (GAP and AESOP-10) was fitted, forcing all predictors into the model regardless of their statistical significance. Rubin's rule was used to combine model parameter estimates across each imputation data-set.

The apparent performance was measured in terms of discrimination (quantified using the C-statistic) and calibration (quantified using the calibration-in-large, calibration slope, ratio of expected to observed number of events (E/O), and calibration plots with smooth calibration curves). The apparent performance was estimated in each imputation data-set and then averaged across all imputation data-sets.

Calibration and discrimination summary statistics were obtained by averaging across imputation data-sets. Calibration plots and decision curve plots are presented for a randomly chosen imputation data-set in the Results section below, and others are given in Supplementary Appendices 8 and 9.

Internal validation was undertaken using bootstrapping of the entire development data-set (accounting for the clustering of participants within studies) to estimate optimism in model performance, and then to derive optimism-adjusted estimates of predictive performance for calibration (e.g. calibration-in-the-large, calibration slope) and discrimination (C-statistic) of predicted risks. Finally, the developed model was adjusted (penalised) by multiplying predictor coefficients by the shrinkage factor (optimism-adjusted calibration slope) and the intercept re-estimated to ensure calibration-in-the-large.

### Sensitivity analyses

Two additional analyses were conducted. First, as the clinical features of FEP at the time of first contact with psychiatric services can be non-specific, formulating a diagnosis of schizophrenia or schizoaffective disorder is not possible in all cases.^42^ Therefore, we conducted the analysis excluding the variable indicating a diagnosis of schizophrenia or schizoaffective disorder and estimated its impact on model performance. Second, performance of both models was checked in each data-set separately, to examine potential heterogeneity of performance across the two cohorts.

### Determining a decision threshold

Decision curve analysis was conducted to explore the potential net benefit of using a prediction model to make treatment decisions (i.e. prescribe clozapine rather than routine antipsychotics), based on a threshold of risk of TRS considered acceptable by clinicians. The decision to treat would depend on the benefits (expected effects) and harms (complications, costs) of the treatment for TRS. If the treatment is effective with minimal costs and risk of complications the preferred threshold is likely to be low, and vice versa. Fifteen clinicians were consulted on their opinion of an appropriate threshold probability at which their decision on treatment decision would change. To facilitate the discussion, the clinicians were presented with two real-life case descriptions (with fictitious names and identifying information), developed together with our patient and expert advisors (Supplementary Appendix 5). The discussion considered the social and clinical implications of clozapine use for young individuals when discussing the balance of expected benefits and harms of clozapine therapy compared with first-line antipsychotics or other treatment options.

### Qualitative analysis

Qualitative data were recorded, transcribed and analysed using a thematic approach.^43^ The concept of information power was operationalised to guide sampling, data collection and analysis.^44^

### Patient and public involvement and engagement (PPIE)

Patient and public involvement and engagement (PPIE) was embedded through a patient and carer advisory group comprising four to six people with psychosis, and a carer who participated in the study design, conduct and analysis and is a co-author (D.S.).

### Ethics approvals

The development of the prognostic model was based on anonymised data from the existing GAP and AESOP-10 cohorts, for which ethical approval is in place. Ethical approval for the qualitative studies was obtained from the Keele University Faculty of Medical and Health Sciences Ethics Committee (Ref: MH-210174). The authors assert that all procedures contributing to this work comply with the ethical standards of the relevant national and institutional committees on human experimentation and with the Helsinki Declaration of 1975, as revised in 2008.

### Consent statement

Written informed consent was obtained from all participants.

## Results

### Study sample

Across the two data-sets there were 1081 participants, 638 of whom had complete outcome data that included 168 (26.3%) with TRS. Baseline characteristics of the two cohorts as well as the combined sample are given in Table 1.

### Prediction model and predictive performance

The main model (model 1) including the initial diagnosis of schizophrenia or schizoaffective disorder is contained in Table 2. The mathematical formula for this model (post-shrinkage) is:
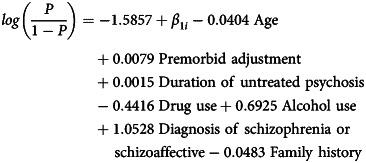
where *β*_1*i*_ ~ *N*(0, (8.09 × 10^−9^)^2^).

**Table 2:** Main model (model 1) after shrinkage including diagnosis of schizophrenia or schizoaffective disorder

Predictor	Coefficient
Age	−0.0404
Premorbid adjustment (NART FSIQ)	0.0079
Duration of untreated psychosis	0.0015
Drug use	−0.4416
Alcohol use	0.6925
Diagnosis of schizophrenia or schizoaffective	1.0528
Family history	−0.0483
Intercept	−1.5857

NART FSIQ, full-scale intelligence quotient (IQ) derived from the National Adult Reading Test.

The shrinkage factor was 0.798. The random effect on the intercept had a standard deviation of 8.09 × 10^−9^.

Table 3 describes the predictive performance of the main model. Apparent discrimination (C-statistic) prior to shrinkage was 0.727 (95% CI 0.723–0.732) and 0.687 after shrinkage (adjustment for optimism), indicating reasonable ability of the model to discriminate between participants with and without TRS after a period of 5–10 years. The apparent calibration performance showed that the ratio of average expected (predicted) risk to average observed risk was close to 1, both before and after adjustment for optimism, indicating good overall agreement between predicted and observed risks.

**Table 3:** Performance of model 1 including diagnosis of schizophrenia or schizoaffective disorder

Statistic	Apparent (before shrinkage)	Optimism adjusted
C-statistic	0.727 (95% CI 0.723 to 0.732)	0.687
Calibration slope	0.999 (95% CI 0.973 to 1.025)	0.798
Calibration-in-the-large	0.0003762 (95% CI 2.93 × 10^−6^ to 0.000749)	0.00584
Expected/observed	0.9998	0.9993

**Fig. 1:**
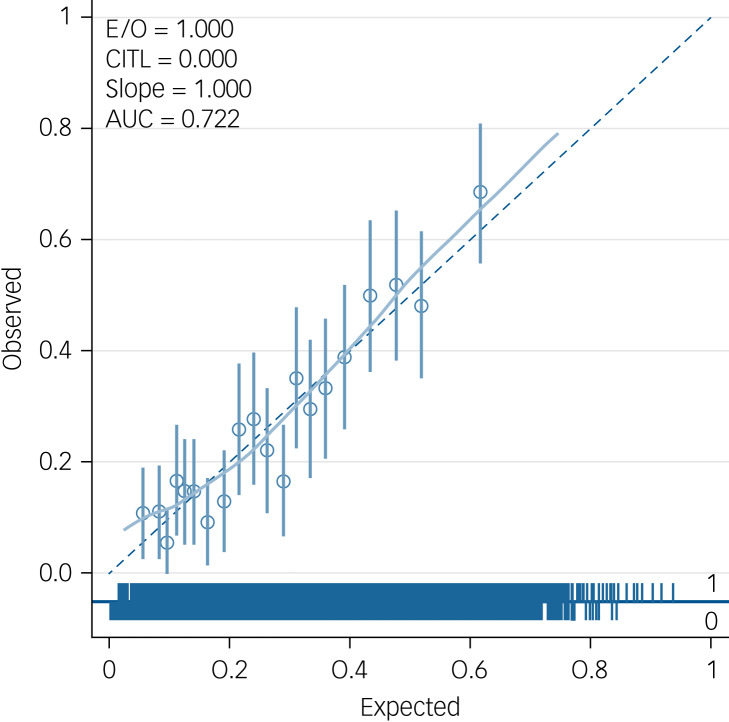
Calibration plot for model 1, including all predictors (based on imputation data-set 31). E/O, expected/observed risk ratio; CITL, calibration-in-the-large; AUC, area under the curve.

Fig. 1 offers an example of a calibration plot prior to shrinkage based on one imputation data-set (data-set 31). The plot confirms a calibration slope close to 1 and predicted risks fairly close to observed risks across the range of observed risk.

### Decision curve analysis

**Fig. 2:**
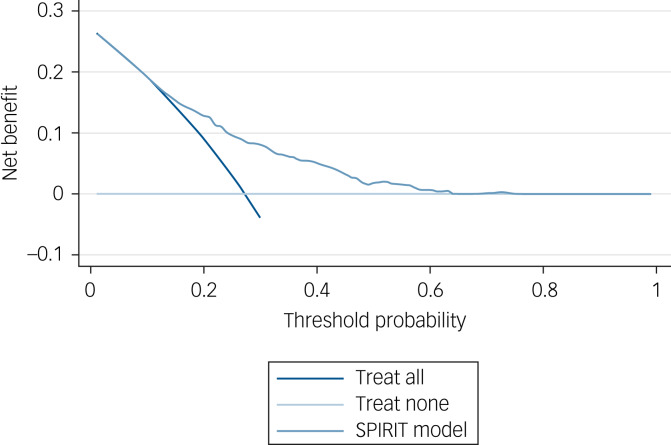
An example plot from the decision curve analysis (from imputation data-set 31). SPIRIT, schizophrenia prediction of resistance to treatment.

During the stakeholder meeting, clinicians preferred a high threshold (at least 50% risk of TRS) for changing treatment from routinely used antipsychotics, illustrating a reluctance to prescribe clozapine. Fig. 2 shows an example (in a random imputation data-set) decision curve for model 1, including competing strategies of treating everyone or treating none. A threshold probability of 0.50 would convey little net benefit, as it would correctly identify only a small number of patients with TRS (close to a net benefit of 0 related to treating no one). Net benefit would be larger when using a risk threshold of around 30% but would expose a larger number of patients to potential harms or burden of treatment. For further information on decision curve analyses, see Supplementary Appendices 4 and 9.

### Sensitivity analyses

As mentioned above, the diagnosis of schizophrenia or schizoaffective disorder may not be possible at the baseline in all individuals with FEP, hence a sensitivity analysis was conducted excluding these diagnoses. Detailed results for the prediction model (referred to as model 2) after excluding an initial diagnosis of schizophrenia or schizoaffective disorder are presented in Supplementary Appendix 3.

As expected, predictive performance for estimating risk of TRS was lower than for the main model. Apparent discrimination (C-statistic) prior to shrinkage was 0.666 (95% CI 0.661–0.671) and 0.621 after adjustment for optimism. The model still calibrated well, with the ratio of average expected (predicted) risk to average observed risk close to 1, and calibration-in-the-large close (−0.002419) after adjustment for optimism, indicating good agreement between predicted and observed risks.

Sensitivity analysis was also undertaken to determine whether the two models based on each data-set performed well in both data-sets. Table 4 presents measures of discrimination for the two models in each of the data-sets, showing similar apparent predictive performance across the two patient samples. As expected, model 1 performs better than model 2 in both data-sets. Discrimination of model 1 appears to be higher in the AESOP data-set (C-statistic 0.736 *v.* 0.718), whereas the reverse is true for model 2 (0.639 *v.* 0.698). The results for calibration (including the calibration plots in Supplementary Appendix 3) indicate that both models may slightly underestimate observed risk of TRS in the GAP data-set, and slightly overestimate TRS risk in the AESOP.

**Table 4:** Measures of discrimination for model 1 and model 2 in each data-set

	GAP (apparent performance)	GAP (optimism adjusted)	AESOP-10 (apparent performance)	AESOP-10 (optimism adjusted)
**Model 1 (including all seven predictors)**				
C-statistic	0.718 (95% CI 0.711 to 0.724)	0.685	0.736 (95% CI 0.730 to 0.742)	0.691
Calibration slope	0.980 (95% CI 0.942 to 1.018)	0.804	1.017 (95% CI 0.981 to 1.052)	0.779
Calibration-in-the-large	−0.0432	−0.0452	0.040	0.030
Expected/observed	1.028	1.028	0.9745	0.981
**Model 2 (excluding schizophrenia diagnosis)**				
C-statistic	0.698 (95% CI 0.691 to 0.705)		0.639 (95% CI 0.632 to 0.646)	
Calibration slope				
Calibration-in-the-large	−0.036039		0.033334	
Expected/observed	1.025		0.9771	

GAP, Genetics and Psychosis cohort; AESOP-10, Aetiology and Ethnicity in Schizophrenia and Other Psychoses.

**Fig. 3:**
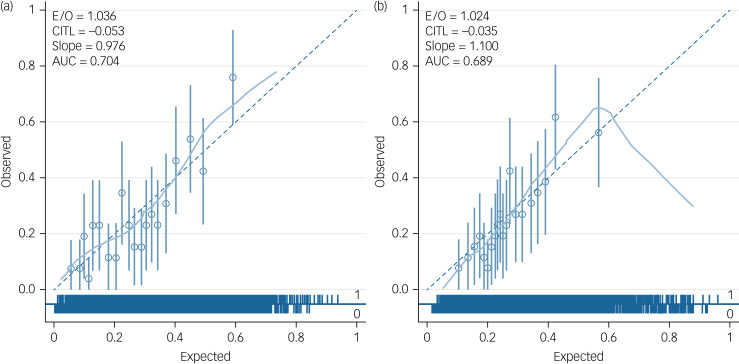
(a) An example calibration plot for the Genetics and Psychosis (GAP) study (from imputation data-set 31). (b) An example calibration plot for the GAP study without schizophrenia diagnosis (from imputation data-set 31). E/O, expected/observed risk ratio; CITL, calibration-in-the-large; AUC, area under the curve.

**Fig. 4:**
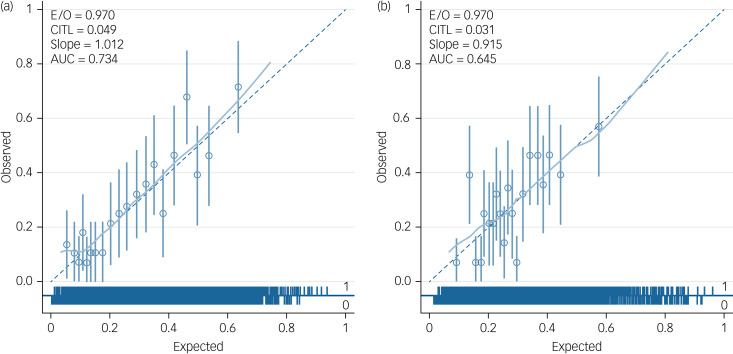
(a) An example calibration plot for the Aetiology and Ethnicity in Schizophrenia and Other Psychoses (AESOP) study (from imputation data-set 31). (b) An example calibration plot for the AESOP study without schizophrenia diagnosis (from imputation data-set 31). E/O, expected/observed risk ratio; CITL, calibration-in-the-large; AUC, area under the curve.

As the development data utilised two data-sets, AESOP and GAP, calibration plots were applied to each data-set to determine how well the model fitted in each data-set. This sensitivity analysis was undertaken to explore whether there was significant miscalibration in either of the data-sets. Examples (from imputation data-set 31, which was randomly selected) of calibration plots for AESOP and GAP are shown in Figs 3 and 4.

Further calibration plots and decision curve plots are presented Supplementary Appendices 8 and 9. Each file is named with the imputation number for all data-sets, and separately for each cohort (labelled as source_1 for GAP and source_2 for AESOP). The calibration plots and decision curve plots are also presented without a diagnosis of schizophrenia.

### Qualitative findings on the clinical application of the model

Eleven clinicians (six psychiatrists; five pharmacists) participated in either a focus group (*n* = 2) or interview (*n* = 3). Six participants were female (five male) with an average age of 47 years (range: 24–64 years) and average length of time in practice of 20 years (range: 8 months to 40 years).

Three main themes were identified from qualitative data: multi-level challenges in identifying and managing TRS, benefits and barriers of the prediction model, and clinical decision aid design preferences. Pseudonymised data are provided in Supplementary Appendix 6 to evidence data interpretation, along with sub-themes.

#### Multi-level challenges in identifying and managing TRS

Common challenges were identified at patient (social stigma, treatment burden, poor adherence), clinician (case complexity, accurate history taking, administrative burden of treatment, making the case for specific treatment), and service and policy levels (service capacity, service availability, treatment guidelines). These challenges established the context for discussion of the prediction model.

#### Benefits and barriers to using the prediction model

All participants recognised the prediction model as a relevant resource with the potential to inform clinical decision-making and case discussion. All members of a multidisciplinary team (i.e. psychiatrists, pharmacists, nurses, care coordinators) were considered likely to benefit from access to the prediction model. However, clinical challenges were identified as potential barriers to implementation and performance of the prediction model, such as the challenge of recording an accurate patient history and confirming a diagnosis of schizophrenia.

Participants acknowledged the importance of using the risk score in conjunction with other information to inform treatment decisions (e.g. history of relapse, hospital admissions, social factors). Participants maintained that treatment guidelines for TRS needed to be adhered to in clinical practice and that a high-risk score should not be perceived as approval to prescribe antipsychotic drugs, such as clozapine, off-label. There was a difference in opinion about whether risk scores for TRS should be shared with patients; concerns were raised about the score potentially posing a barrier to communication, contributing to stigma and affecting attitudes to treatment.

#### Clinical decision aid design preferences

Participants identified opportunities to inform design of a clinical decision aid based on the model and support implementation. Access to published evidence reporting the development and validation of the prediction model was expected by participants to demonstrate robustness. Participants also requested a package of training to guide appropriate use, inform interpretation of risk scores and enable objective categorisation of risk – particularly as the list of predictors were commonly presented in practice. Participants advised that an online or mobile phone application format of the fully developed and validated model and its interpretation would help in clinical practice.

## Discussion

We developed a prediction model for estimating the risk of TRS based on seven readily available clinical predictors that are predominantly routinely collected in people presenting with FEP. The model performed promisingly in internal validation and had reasonable ability to discriminate between participants with and without TRS at 5- or 10-year follow-up (C-statistic 0.687 after adjustment for optimism) and had good calibration (expected/observed ratio: 0.999; calibration-in-the-large: 0.000584). The performance of the SPIRIT prediction model is consistent with three recently published studies^45–47^ that reported prediction models for TRS and reported C-statistics of 0.59, 0.60 and 0.70 respectively. Only one of these studies used prospective FEP-cohort data to develop the prediction model.^45^ The other studies used routinely recorded information from clinical notes from early intervention in psychosis services^47^ and from electronic health records using the Clinical Record Interactive Search (CRIS) system for retrieving information on treatment resistance and its predictors.^46^ The latter approach has limitations, including the use of clozapine as proxy for the definition of the main outcome of treatment resistance, given the long delays often associated with using clozapine after development of treatment resistance.

### Strengths

Our study used two well-defined cohorts with adequate sample size for the model development approach, and defined treatment resistance in terms of criteria required for clozapine use as well as non-remission of psychosis based on a consensus definition.^27^ We followed the PROGRESS framework for developing and testing the model, prospectively published the protocol in open access^15^ and reported using the TRIPOD checklist. We used a mixed-methods design to incorporate also an exploration of acceptability and we had robust patient and public involvement in all stages of research (to enhance the potential for application of the model in clinical practice) and in model development, which has not been reported in other studies.

The qualitative methods used in the study add further methodological value and key data to inform the translation of prediction models for psychosis into clinical practice. The sample size may seem small and limited to two geographical regions in the UK, which may limit transferability. However, the objective for the qualitative study was narrow, sampling specificity was limited to two NHS trust settings and specific professionals, and dialogue during data collection was strong (further enriched by focus group methods), thus satisfying several dimensions of information power that guide the sample size determination in qualitative studies.^44^

### The decision threshold and concerns about clozapine

Consultation with clinicians showed that they preferred a high threshold (indicative of at least 50% risk of TRS) for changing treatment from routinely used antipsychotics, illustrating a reluctance to prescribe clozapine. At this threshold the model would not have significant net benefit, as it would correctly identify only a small number of patients with TRS. The model would have clinical utility at a threshold of 30% or lower. Further research on this issue is needed.

The decision threshold discussions with clinicians revealed that the early use of clozapine is still considered a high-risk strategy and probably highlights the need for better education and training about the use of clozapine in early psychosis.^48,49^ Including a clinical decision tool in the decision process to start clozapine will require regulatory approvals and full impact analysis studies, such as cluster RCTs, or large comparative observational studies. However, after external validation, our clinical prediction model could inform the stratification of patients at higher-than-average risk of developing TRS. This will represent a significant advance in the current state of clinical practice, where the use of clozapine is delayed for 5–10 years, and is likely to be a highly cost-effective strategy. In a modelling study, Jin et al (2019)^50^ used a hypothetical scenario to estimate the benefit of such a prediction model. They showed that using a prediction model (that hypothetically could allow the use of clozapine after patients failed to respond to one antipsychotic instead of the current standard of offering clozapine only after failure of two antipsychotics) would result in an improvement of 0.10 quality-adjusted life years (QALYs). This could reduce annual healthcare costs by £7363 per person. The analysis also suggested that implementing a predictive model for this purpose would be more cost-effective than treatment as usual even if it only accurately identified 6% of treatment-resistant patients and 50% of non-treatment-resistant patients. The closer monitoring of those at high risk will also help to implement evidence-based psychological and social interventions to enhance recovery.

### Barriers to implementation of prediction models

The lack of implementation of prediction models in clinical practice is recognised as a universal problem in psychiatry and a review of 89 studies identified only one implementation study.^51–53^ We limited our model to clinical predictors and explored the clinical application of the model by involving patients, carers and clinicians, who highlighted the potential barriers in clinical translation of the prediction model (Supplementary Appendix 6). The potential barriers included confirming schizophrenia diagnosis, stigma in using the term ‘TRS’ and lack of relevant training. All participants agreed that the risk score from the model could be used in conjunction with other information (e.g. history of relapse, hospital admissions, social factors) to inform treatment decisions. Importantly they highlighted the need for a package of training to guide appropriate use, inform interpretation of risk scores and enable objective categorisation of risk. These barriers to implementation and the training requirements need to be addressed in future studies.

### Limitations

Limitations of the study include the presence of missing data, lack of external validation of the prediction model and limited assessment of generalisability, as the participants recruited to the cohorts were predominantly from urban populations in a high-income country. Participants in both cohorts were recruited prior to 2010 and management of FEP is likely to have changed since that time. It is imperative that the model is externally validated in the newer cohorts**.** Also, the model included only seven potential predictors. We aimed to have a model that could predict the risk of TRS in routine clinical practice and predictors were therefore limited to easily assessed clinical characteristics. It was not possible to include well-known determinants of outcome such as non-adherence to antipsychotic treatment and initial response to antipsychotic treatment in this model as these predictors are not available at the time of initial assessment. A systematic review showed that psychiatry prediction models encompassing biomarkers or a large number of predictors (which may be more prone to overfitting issues) are not necessarily better.^53^ Similarly, a prediction model based on polygenic risk score was of little use for early identification of TRS and not of clinical utility.^54,55^

### Implications

We demonstrated that a prediction model based on a small number of predominantly routinely collected clinical variables that can be assessed in routine clinical practice can potentially inform the identification of patients who are likely to develop TRS. We used the NART^37^ to assess premorbid functioning and this instrument is not used in routine clinical practice. However, clinical features such as premorbid functioning are routinely assessed in clinical history taking in early intervention in psychosis services^56^ and it will be important to examine how the measurement of these variables based on clinical assessment alone affects the performance of the prediction model in future studies. The clinicians showed willingness to use the model but had a high threshold for changing current clinical practice. Several steps are necessary before the model can be considered for clinical use. These include testing of the external validity of the prognostic model (regarding both 5- and 10-year outcomes) to examine calibration across a range of settings and regions, and further assessment of decision thresholds and clinical utility at those thresholds. The response to treatment in schizophrenia varies depending on the stage of illness^57^ and future research may need to incorporate stage of illness into the model, or test and potentially update the model for such patient groups, incorporating, for example, response to initial treatment.

## Supporting information

Farooq et al. supplementary material Farooq et al. supplementary material

## Data Availability

In accordance with the UK Research Council's Common Principles on Data Policy, we are working towards making data supporting this study available. Research materials and analytic codes are available from the corresponding author, S.F., on reasonable request.
