# Supplementary material for: Development and initial evaluation of a clinical prediction model for risk of treatment resistance in first-episode psychosis: Schizophrenia Prediction of Resistance to Treatment (SPIRIT)
Source: Br J Psychiatry. 2024 Sep;225(3):379–88. doi: 10.1192/bjp.2024.101 (PMC11536189; doi:10.1192/bjp.2024.101)
Supplement: Farooq et al. supplementary material [file S0007125024001016sup001.docx]

**Table of Contents**

Appendix 1 2

TRIPOD checklist: Prediction model development and validation 2

Appendix 2 4

The identification of predictive variables most associated with treatment resistance schizophrenia 4

Appendix 3 6

The model 2: Performance and sensitivity analysis 6

Appendix 4 11

The model 1: Decision curve analysis 11

Appendix 5 12

Case vignettes used for discussing the prediction model with clinicians and service users 12

Appendix 6 15

SPIRIT qualitative findings 15

Appendix 7 19

Description of predictor variables 19

Appendix 8 22

All calibration plots 22

Appendix 9 23

All decision curve analysis plots 23

Appendix 10 24

SPIRIT protocol paper 24

# **Appendix 1**

## **TRIPOD checklist: Prediction model development and validation**

| **Section/Topic** | **Item** |  | **Checklist Item** | **Page** |
| --- | --- | --- | --- | --- |
| **Title and abstract** | | | | |
| Title | 1 | D;V | Identify the study as developing and/or validating a multivariable prediction model, the target population, and the outcome to be predicted. | 1 |
| Abstract | 2 | D;V | Provide a summary of objectives, study design, setting, participants, sample size, predictors, outcome, statistical analysis, results, and conclusions. | 2 |
| **Introduction** | | | | |
| Background and objectives | 3a | D;V | Explain the medical context (including whether diagnostic or prognostic) and rationale for developing or validating the multivariable prediction model, including references to existing models. | 3 |
|  | 3b | D;V | Specify the objectives, including whether the study describes the development or validation of the model or both. | 3 |
| **Methods** | | | | |
| Source of data | 4a | D;V | Describe the study design or source of data (e.g., randomized trial, cohort, or registry data), separately for the development and validation data sets, if applicable. | 3 |
|  | 4b | D;V | Specify the key study dates, including start of accrual; end of accrual; and, if applicable, end of follow-up. | 4 |
| Participants | 5a | D;V | Specify key elements of the study setting (e.g., primary care, secondary care, general population) including number and location of centres. | 4 |
|  | 5b | D;V | Describe eligibility criteria for participants. | 4 |
|  | 5c | D;V | Give details of treatments received, if relevant. | 4 |
| Outcome | 6a | D;V | Clearly define the outcome that is predicted by the prediction model, including how and when assessed. | 5 |
|  | 6b | D;V | Report any actions to blind assessment of the outcome to be predicted. | NA |
| Predictors | 7a | D;V | Clearly define all predictors used in developing or validating the multivariable prediction model, including how and when they were measured. | 5,6 |
|  | 7b | D;V | Report any actions to blind assessment of predictors for the outcome and other predictors. | NA |
| Sample size | 8 | D;V | Explain how the study size was arrived at. | 6 |
| Missing data | 9 | D;V | Describe how missing data were handled (e.g., complete-case analysis, single imputation, multiple imputation) with details of any imputation method. | 6 |
| Statistical analysis methods | 10a | D | Describe how predictors were handled in the analyses. | 5,6 |
|  | 10b | D | Specify type of model, all model-building procedures (including any predictor selection), and method for internal validation. | 6,7 |
|  | 10c | V | For validation, describe how the predictions were calculated. | 6,7 |
|  | 10d | D;V | Specify all measures used to assess model performance and, if relevant, to compare multiple models. | 6,7 |
|  | 10e | V | Describe any model updating (e.g., recalibration) arising from the validation, if done. | NA |
| Risk groups | 11 | D;V | Provide details on how risk groups were created, if done. | NA |
| Development vs. validation | 12 | V | For validation, identify any differences from the development data in setting, eligibility criteria, outcome, and predictors. | 6,7 |
| **Results** | | | | |
| Participants | 13a | D;V | Describe the flow of participants through the study, including the number of participants with and without the outcome and, if applicable, a summary of the follow-up time. A diagram may be helpful. | 5 |
|  | 13b | D;V | Describe the characteristics of the participants (basic demographics, clinical features, available predictors), including the number of participants with missing data for predictors and outcome. | 5,6 |
|  | 13c | V | For validation, show a comparison with the development data of the distribution of important variables (demographics, predictors and outcome). | 6 |
| Model development | 14a | D | Specify the number of participants and outcome events in each analysis. | 5,6 |
|  | 14b | D | If done, report the unadjusted association between each candidate predictor and outcome. | NA |
| Model specification | 15a | D | Present the full prediction model to allow predictions for individuals (i.e., all regression coefficients, and model intercept or baseline survival at a given time point). | 8,9 |
|  | 15b | D | Explain how to the use the prediction model. | 8,9 |
| Model performance | 16 | D;V | Report performance measures (with CIs) for the prediction model. | 8,9 |
| Model-updating | 17 | V | If done, report the results from any model updating (i.e., model specification, model performance). | NA |
| **Discussion** | | | | |
| Limitations | 18 | D;V | Discuss any limitations of the study (such as nonrepresentative sample, few events per predictor, missing data). | 15,16 |
| Interpretation | 19a | V | For validation, discuss the results with reference to performance in the development data, and any other validation data. | 15 |
|  | 19b | D;V | Give an overall interpretation of the results, considering objectives, limitations, results from similar studies, and other relevant evidence. | 15,16 |
| Implications | 20 | D;V | Discuss the potential clinical use of the model and implications for future research. | 16,17 |
| **Other information** | | | | |
| Supplementary information | 21 | D;V | Provide information about the availability of supplementary resources, such as study protocol, Web calculator, and data sets. | 17 |
| Funding | 22 | D;V | Give the source of funding and the role of the funders for the present study. | 18 |

*Items relevant only to the development of a prediction model are denoted by D, items relating solely to a validation of a prediction model are denoted by V, and items relating to both are denoted D;V. We recommend using the TRIPOD Checklist in conjunction with the TRIPOD Explanation and Elaboration document.

#

**Appendix 2**

## **The identification of predictive variables most associated with treatment resistance schizophrenia**

We used an iterative approach to identifying candidate predictors including (i) a review of relevant literature, [22-23,41-43, 24,40]. Additional focused searches (Medline, EMBASE, PsychINFO), and (ii) consultation with our expert and patient advisory group.

The literature review, consultation with expert advisory group and clinicians advisory group produced a list 34 variables. There was some overlap between suggested variables (e.g. baseline symptoms severity and baseline severity of positive and negative symptoms suggested separately as different variables. After removing these overlaps following list of 23 variables was generated.

Once the set of candidate predictors was established, we had series of meetings with our clinical expert and patient advisory groups to decide on those to prioritise for inclusion in the prognostic model development (as the total will need to consider sample size restrictions in order to reduce potential for overfitting). These meetings were held use face-to-face (patient advisory group) and videoconference (clinical expert advisory group) meetings, These meetings were facilitated by an experienced qualitative researcher (TK), clinical member of the study team (SF) and – for the patient advisory group – by the PPIE team member (DS). The advisory groups were presented with a list of all candidate predictors available in the individual participant data (IPD) from two datasets (see below), along with a description of how the predictors have been measured and a brief summary of the existing evidence for their association with resistance to anti-psychotic treatment. Discussions were audio-recorded, and written reports drafted summarising feedback and advice that ranks the predictors by their perceived prognostic importance. The agreed list of candidate predictors will then be taken forward for model development (objective ii).

The results of this process resulted in top ten candidate predictors (given on 1-10 in the list below) to be considered in the development of the proposed prediction model. From these top 10 candidate predictors, we took a subset that that were available in both data sources for the model development. Additional predictors assumed to be important by our experts and patient advisors but not available in the two data sources can be considered for future updates or improvements of the model, if needed, as new data become available.

| **Predictor** |
| --- |
| 1. Response to antipsychotics after 2 weeks of treatment |
| 1. Development of tardive dyskinesia |
| 1. Gender: Male gender associated with poorer outcome |
| 1. Ethnic origin: Afro Caribbean associated with poorer outcome |
| 1. Comorbidity such as substance use, personality disorder, ADHD, ASD. |
| 1. Educational level: Lower educational level associated with treatment resistance |
| 1. Family history of psychosis |
| 1. Marital status. Single status associated with treatment resistance |
| 1. Increased genetic burden/family history of psychosis |
| 1. Premorbid adjustment and premorbid functioning |
| 1. Good insight is associated with recovery and less treatment resistance |
| 1. Cognitive impairment |
| 1. Neurobiological and Neurological soft signs |
| 1. Cognitive performances and disorganized symptoms |
| 1. Diagnosis of schizophrenia (instead of psychotic depression or psychotic mania) |
| 1. Age of onset of the Illness: Younger age at onset predicts poor response and treatment resistance |
| 1. Duration of Untreated Psychosis (DUP) |
| 1. Longer Illness duration predicts poor response to treatment and treatment resistance. (The longer duration of illness includes the total duration of illness of patient have. This will include duration before treatment and after treatment when symptoms are not resolved |
| 1. Baseline total symptom severity |
| 1. Non-adherence to antipsychotic treatment |
| 1. Having spent more than 30 bed-days in a psychiatric hospital in the year before diagnosis |
| 1. Paranoid subtype diagnosis |
| 1. Baseline motor function |

#

# **Appendix** **3**

## **The model 2: Performance and sensitivity analysis**

**Model 2 – not including diagnosis of schizophrenia or schizoaffective disorder**

The model not including diagnosis of schizophrenia or schizoaffective is contained in the table below and written as a mathematical formula below the table.

| **Predictor** | **Coefficient** |
| --- | --- |
| Age | -0·0376 |
| Premorbid adjustment (nart_fiq) | 0·0028 |
| Duration of untreated Psychosis | 0·0016 |
| Drug use | -0·3953 |
| Alcohol use | 0·6529 |
| Family history | 0·0325 |
| Intercept | -0·5225 |

The random effect on the intercept had a standard deviation of 7·45e^-9^.

The written mathematical equation for model 2 is:

$$log\left( \frac{P}{1-P} \right)=-0\cdot5225+\beta_{1i}-0\cdot0376 Age+0\cdot0028 Premorbid adjustment+0\cdot0016 Duration of untreated psychosis-0\cdot3953 Drug use+0\cdot6529 Alcohol use+0\cdot0325 Family history$$

where $\beta_{1i}\sim N(0, \left( 7\cdot45e^{-9} \right)^{2}$)

**The model performance for model 2 is:**

| **Statistic** | **Apparent (before shrinkage)** | **Optimism adjusted** |
| --- | --- | --- |
| **C statistic** | 0·666  (95% C.I.: 0·661 to 0·671) | 0·621 |
| **Calibration slope** | 0.999  (95% C.I.: 0.964 to 1.034) | 0.721 |
| **Calibration-in-the-large** | 0·0004096 | -0·002419 |
| **Expected/**  **Observed** | 0·9997 | 1·0043 |


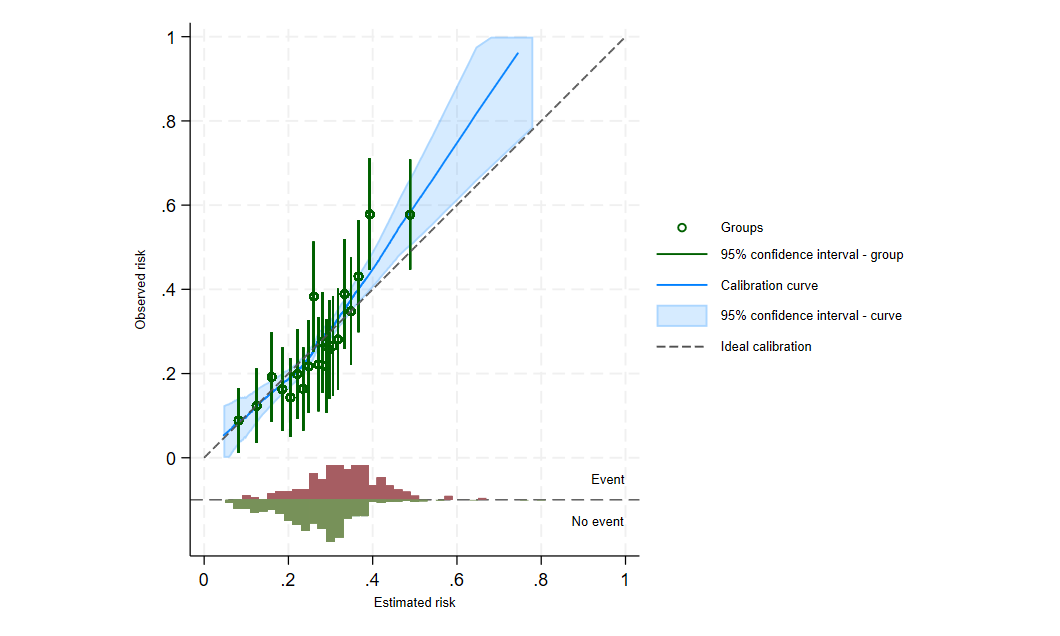


The calibration plot for the apparent model 2 (model excluding schizophrenia diagnosis) averaged across all imputation datasets


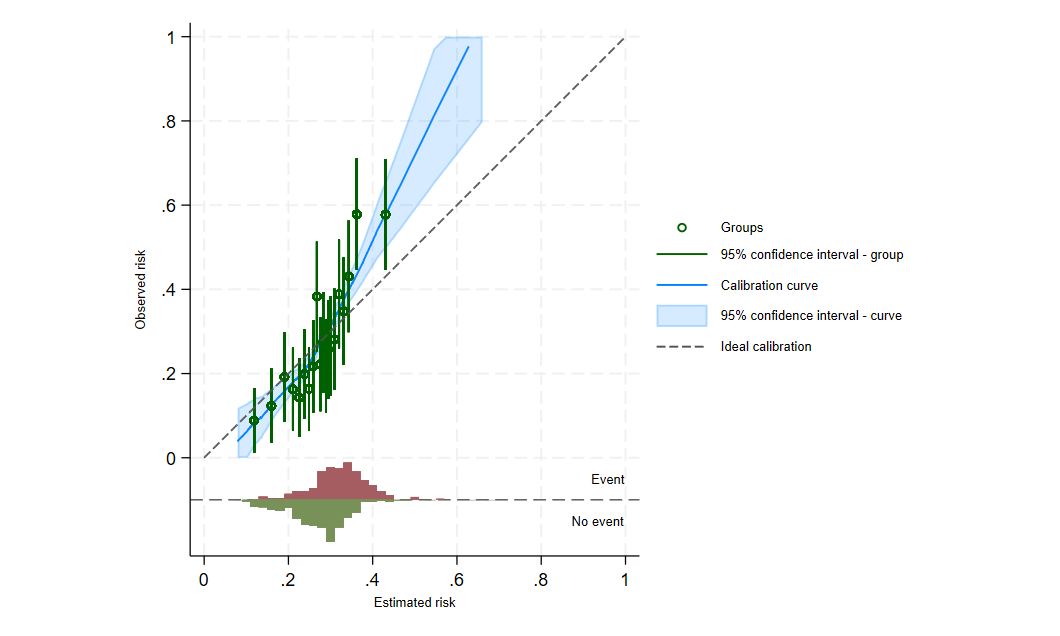


The calibration plot for the optimism adjusted model 2 (model excluding schizophrenia diagnosis) averaged across all imputation datasets

**Model 2 Decision curve analysis for imputation 23**
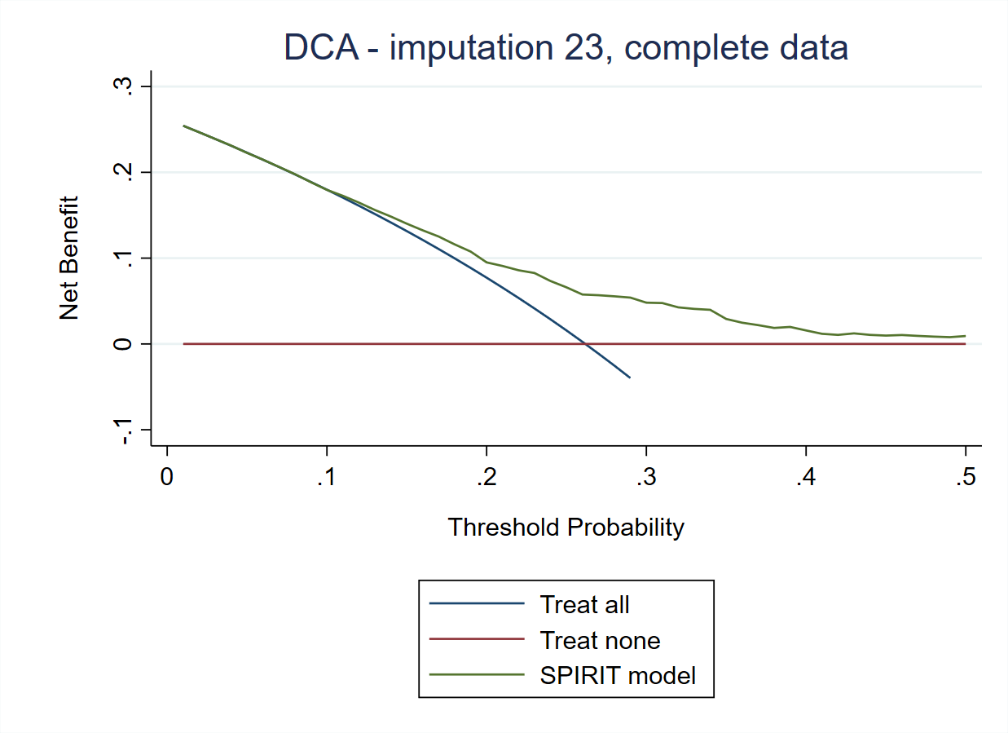


**Sensitivity analysis for Model 2**

Sensitivity analysis was undertaken to determine whether the model performs well in both datasets. The model was applied in both datasets separately and model performance in each dataset was analysed.

**Calibration plots for GAP and AESOP for Model 2**


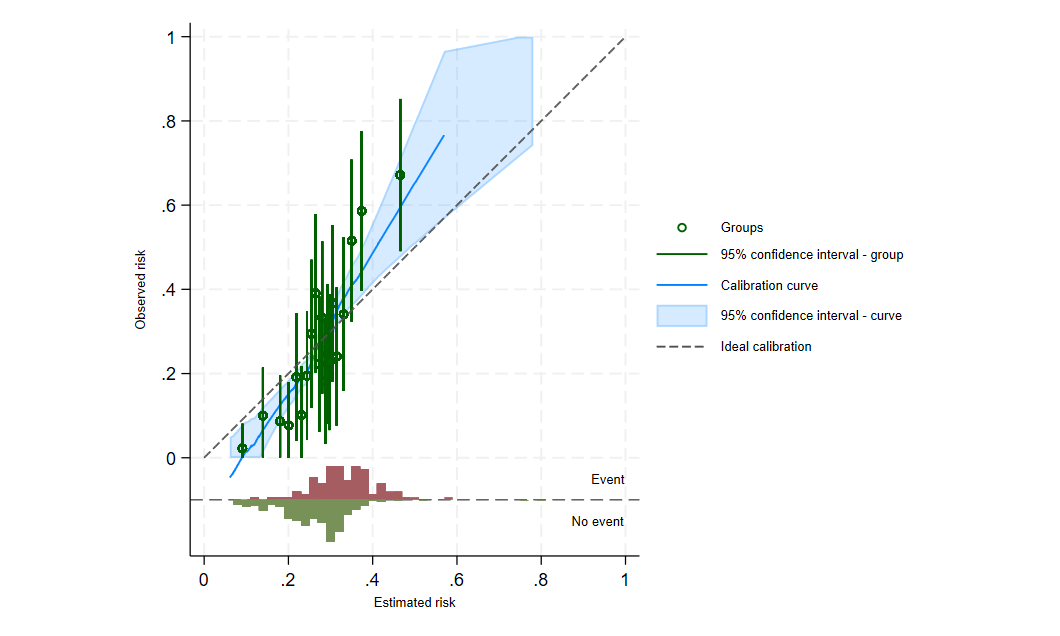


The calibration plot for the apparent model 2 (model excluding schizophrenia diagnosis) for the GAP study averaged across all imputation datasets


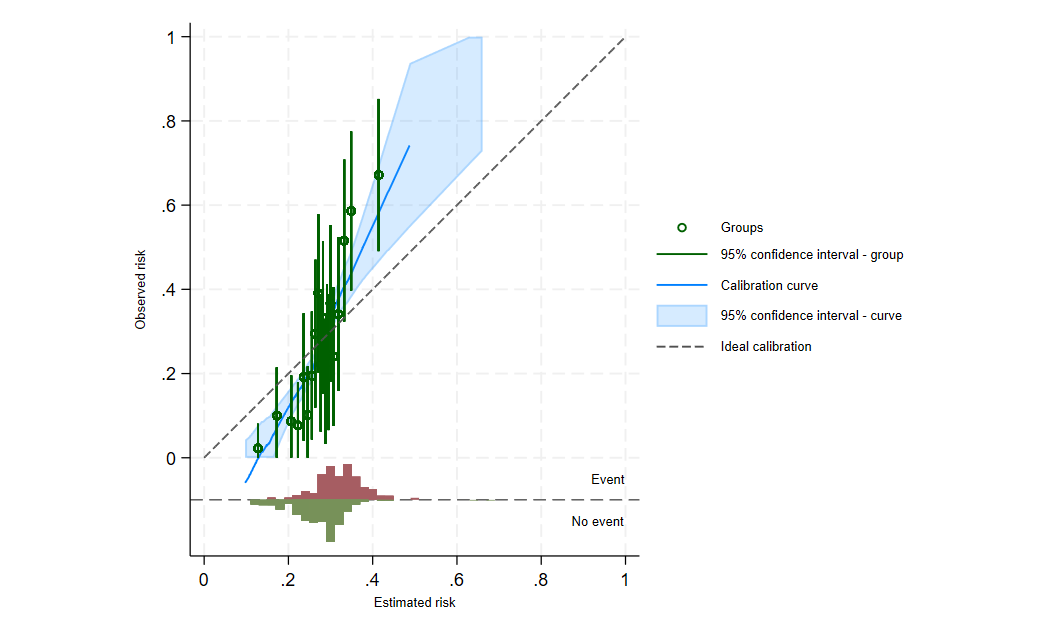


The calibration plot for the optimism adjusted model 2 (model excluding schizophrenia diagnosis) for the GAP study averaged across all imputation datasets


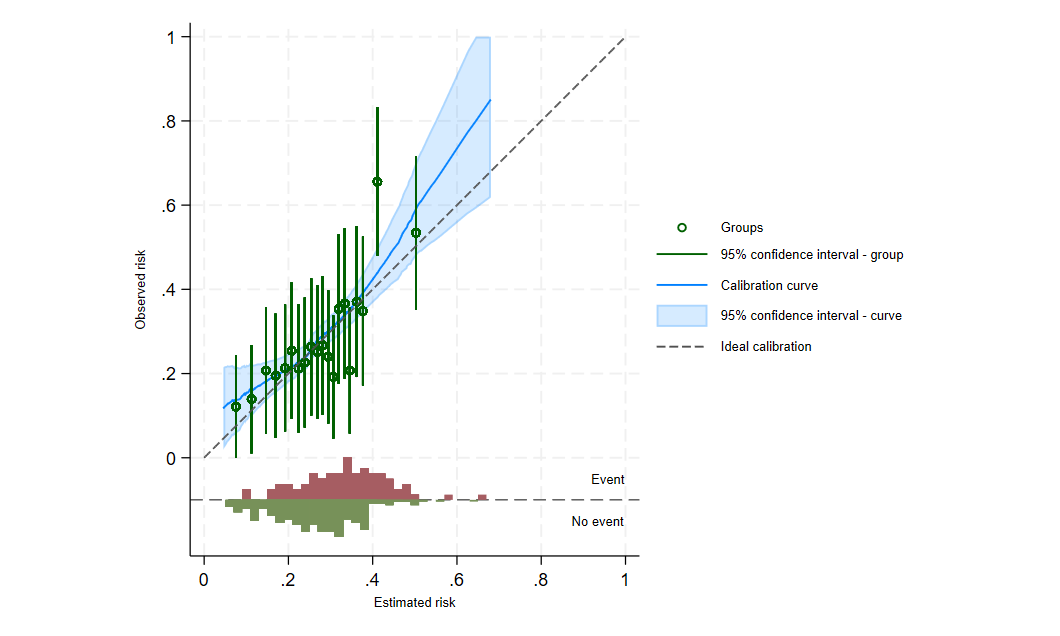


The calibration plot for the apparent model 2 (model excluding schizophrenia diagnosis) for the AESOP studyaveraged across all imputation datasets

The calibration plot for the optimism adjusted model 2 (model excluding schizophrenia diagnosis) for the AESOP study averaged across all imputation datasets

**Model 2 performance**

| **Statistic** | **GAP (Apparent performance)** | **GAP (Optimism adjusted)** | **AESOP (Apparent performance)** | **AESOP (Optimism adjusted)** |
| --- | --- | --- | --- | --- |
| **C statistic** | 0·698  95% CI: 0·691 to 0·705 | 0.650 | 0·639  95% CI: 0·632 to 0·646 | 0.587 |
| **Calibration slope** | 1.244  (95% CI: 1.189 to 1.299) | 0.886 | 0.814  (95% CI: 0.769 to 0.860) | 0.530 |
| **Calibration-in-the-large** | -0·036 | -0.054 | 0·033 | 0.011 |
| **Expected/**  **Observed** | 1·025 | 1.037 | 0·977 | 0.993 |

**Decision curve analysis for Model 2**

The decision curve analysis from imputation dataset 23 for the GAP study


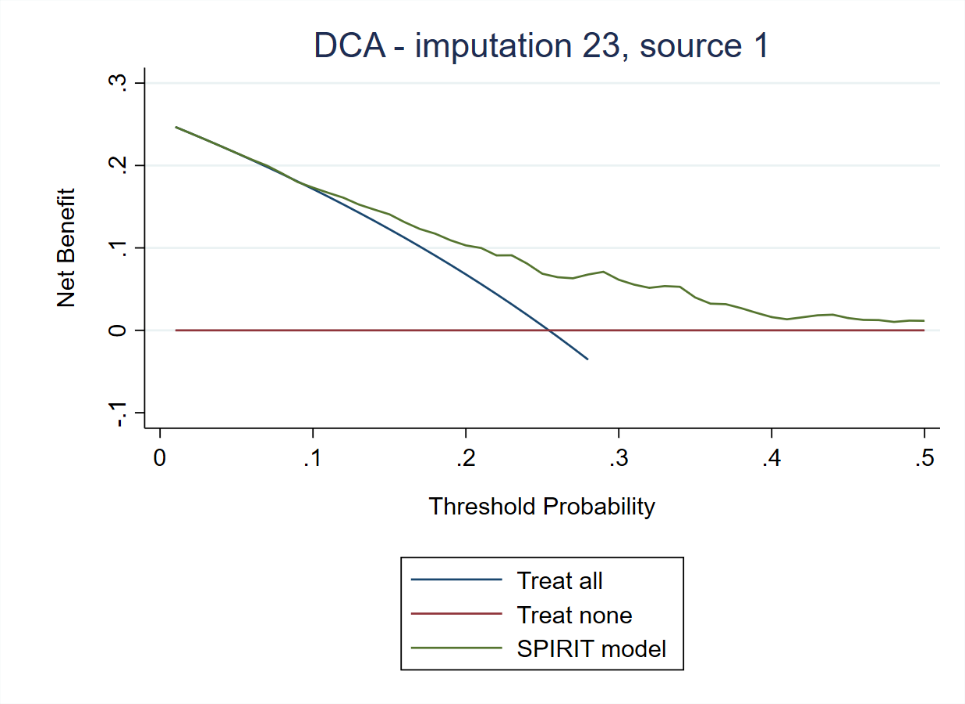


The decision curve analysis from imputation dataset 23 for the AESOP study

# **Appendix 4**

**Decision curve analysis for Model 1 (in both datasets)**

The decision curve analysis based on threshold as discussed above (at least 50% risk of TRS) for changing treatment from routinely used antipsychotics, the results from imputation dataset 31 for the GAP and AESOP study are produced below:


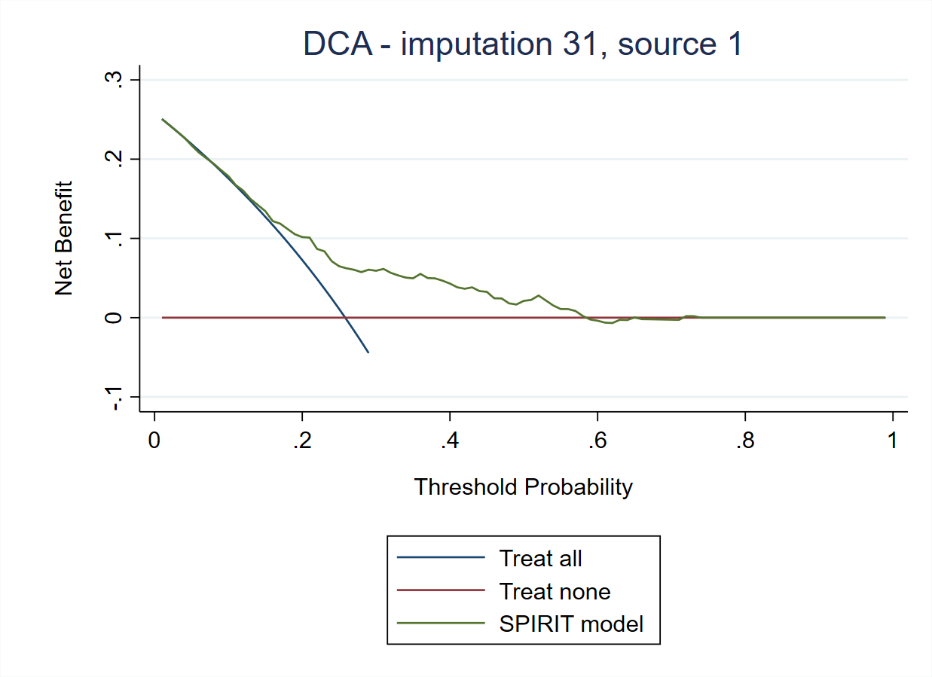


**Figure 5:** The decision curve analysis from imputation dataset 31 for the GAP study


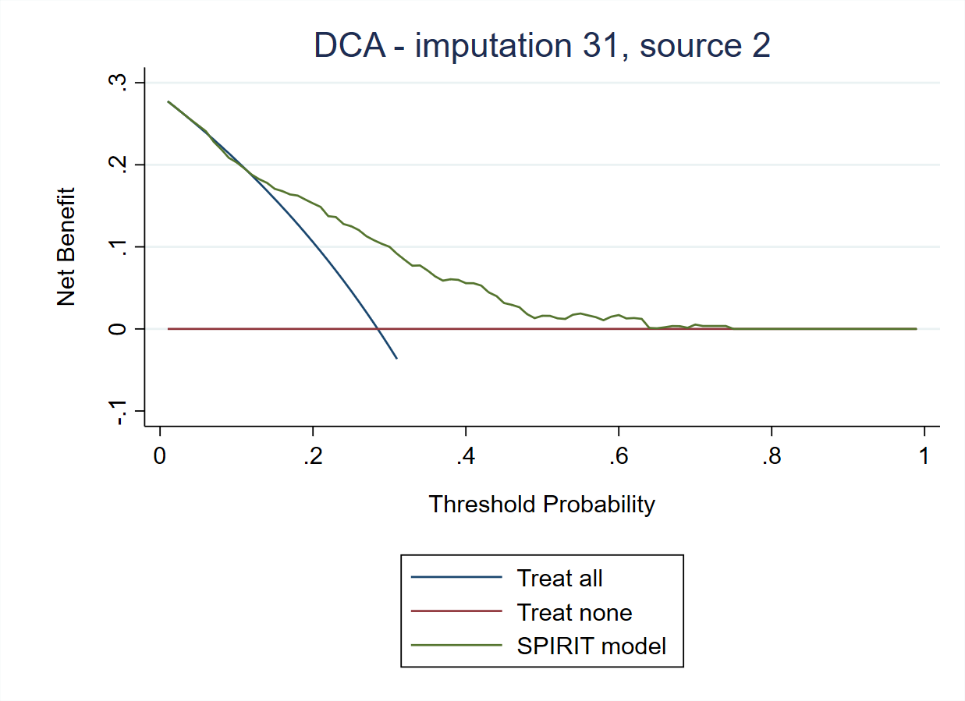


**Figure 6:** The decision curve analysis from imputation dataset 31 for the AESOP study

#

**Appendix 5**

## **Case vignettes used for discussing the prediction model with clinicians and service users**

**DISCLAIMER**

We will be discussing a study that aims to develop a risk prediction model that can be used in clinical practice for predicting the likelihood of developing treatment resistant Schizophrenia (TRS), who present with First Episode Psychosis (FEP). We will be presenting a hypothetical case and would like to know your views regarding this risk prediction model. This study or the model we are investigating is in very early stages of development. The information we discuss today does NOT represent any clinical advice and the model we are discussing today does NOT predict the onset of TRS in people with FEP. This information MUST NOT be used in any way for making any clinical decisions in your clinical practice.

**Case vignette 1: Jason**

**Jason** is 19 years old. He was hospitalised after Police attended his flat. The neighbours complained that he threatened them with a knife. He was shouting at people in street and blamed them that they are stealing from his flat.

At psychiatric assessment, Jason was found to be a poorly groomed. He appeared to be inattentive and preoccupied. It was difficult to follow her speech, as he was constantly talking about organised crime and gangs. The family members revealed Jason behaviour has changed over last two years but Jason was adamant that there was nothing wrong with him. His parents noted that his behaviour became increasingly odd. He stopped seeing his friends and no longer seemed to care about his appearance. He began wearing the same clothes each day and seldom bathed. Jason’s sister said that he had often seen him mumbling quietly to herself and at times he seemed to be talking to people who were not there. They found that his room was full of clutter. He would not dispose waste from the room, believing that this would harm the environment. Further assessment in the psychiatric unit confirmed that Jason had a diagnosis of schizophrenia.

His family suspected that Jason would use cannabis most days in a week and also started drinking heavily soon after leaving the school.

Jason had symptoms for almost two years but treatment could not be started due to lack of willingness by Jason to seek treatment. He was referred to a psychologist but Jason stopped seeing the her after a couple of appointments, as he found that the psychologist had attended the same college as her. He believed that the psychologist could be part of the college ‘gang’ and would involve him in the organised crime.

Jason father and a paternal uncle had a psychotic illness. Jason’s grandmother had had a serious mental illness and had received regular psychiatric treatment for over 30 years.

The family revealed that Jason is generally a shy person and will spend most of his time in in his room. He was suspended from the school few times as he blamed different teachers for not paying enough attention to him. He blamed them for his poor grades sometimes accusing them for favouring his other class fellows.

**Jason risk of developing treatment resistance schizophrenia**

We analysed data from two large cohorts of **first episode psychosis (FEP)** and examined the risk of developing **treatment resistant schizophrenia (TRS).** As you may be aware the use of Clozapine is delayed in clinical practice for several years. The research also shows that generally more than two courses of antipsychotics are used before clozapine is started, despite the NICE guidelines that advice switching to Clozapine, once a patient does not respond to two antipsychotics.

Using evidence from previous research and expert advice we identified a series of clinical features that are already present when patients first present to psychiatric service and that are associated with the likelihood of developing TRS. Based on these clinical features we developed a risk prediction model for predicting the onset of TRS at the time of the first contact with services. The purpose of this model is to estimate the risk of TRS in people with first episode psychosis, and identify those people who are most likely to develop TRS at the earliest possible stage and provide them with an effective intervention such as Clozapine, or other forms of therapies to improve their outcomes.

**A**. Before we can tell you about the model we developed, I have few questions for you:

1. Reviewing the Jason case, what do you think are clinical features in Jason history that would predict the high likelihood of developing TRS. Can you please identify these clinical features?
2. After participants have identified the clinical features, they will be asked to rank these clinical features in order of importance regarding the likelihood of TRS.
3. What do you think is Jason risk of developing TRS; mild moderate, high.
4. What are your reasons for placing Jason in the risk category you assigned? (For each participant we will ask him/her for the reasons for the risk category).

**B**. We are particularly interested in determining a threshold for the risk prediction that clinicians are likely to use in clinical practice i.e. a threshold of the risk above which a clinician would consider using treatments such as Clozapine instead of first generation antipsychotics.

In a **hypothetical** situation as that depicted in Jason case, if the model accurately predicts the likelihood of Jason developing TRS with a 70% accuracy, will you:

1. Consider using a treatment such as Clozapine, instead of a first line antipsychotics
2. Would the use of a decision treatment tool like this change your practice at all?
3. What in your view would be the best threshold at which you would consider using an alternative treatment strategy such as clozapine?

**Case vignette 2: Kelly**

Kelly is 35 years’ old. She first presented to the GP complaining that she has been feeling very anxious and have lost interest in all her activities for the last two months. She had lost appetite and was worried that her food tastes differently. She complained that this started after a party with her friends when she felt that they are all talking about her. Kelly was referred to a community mental health team which Kelly reluctantly agreed.

During the psychiatric assessment, Kelly revealed that she had been hearing voices for the past two months. She felt frightened due to these voices and would spend most of her time outside her home as she did not feel safe at home. On further assessment, it was revealed that Kelly had not been sleeping well and had poor personal hygiene. She appeared anxious and apprehensive, paced to and fro during the consultation. Kelly complained that she heard people talking about her. She was therefore suspicious and felt uneasy with others. She also admitted hearing voices that commanded her not to disclose her condition to anyone. Kelly thought that she had some psychological problem, probably anxiety due to excessive work she had to do recently. She denied feeling hopelessness or having suicidal ideas. She was however very worried about people talking and commenting about her and wanted some help about this

Kelly lives with her mother and sister but did not want them to be contacted. She had a boyfriend who was studying at a local university. Kelly said that her boyfriend is very supportive and had advised her to seek help and therefore she contacted the GP.

No family history of mental or physical illness was noted. Kelly was a generally happy person, who enjoyed company of her friends and was liked by her colleagues at work. She had good academic record and had a degree in finance with distinction. She worked as finance manager in a local firm.

There was no history of substance abuse

After a thorough psychiatric assessment, Kelly was diagnosed as suffering from paranoid schizophrenia and agreed to take medications under the supervision of the home treatment team. Her boyfriend agreed to support her and work with the community mental health team.

**Kelly’s risk of developing treatment resistance schizophrenia:** We analysed data from two large cohorts of **first episode psychosis (FEP)** and examined the risk of developing **treatment resistant schizophrenia (TRS).** As you may be aware the use of Clozapine is delayed in clinical practice for several years. The research also shows that generally more than two courses of antipsychotics are used before clozapine is started, despite the NICE guidelines that advice switching to Clozapine, once a patient does not respond to two antipsychotics.

Using evidence from previous research and expert advice we identified a series of clinical features that are already present when patients first present to psychiatric service and that are associated with the likelihood of developing TRS. Based on these clinical features we developed a risk prediction model for predicting the onset of TRS at the time of the first contact with services. The purpose of this model is to estimate the risk of TRS in people with first episode psychosis, and identify those people who are most likely to develop TRS at the earliest possible stage and provide them with an effective intervention such as Clozapine, or other forms of therapies to improve their outcomes.

**A**. Before we can tell you about the model we developed, I have few questions for you:

1. Reviewing the Kelly case, what do you think are clinical features in Kelly history that would predict the high likelihood of developing TRS. Can you please identify these clinical features?
2. After participants have identified the clinical features, they will be asked to rank these clinical features in order of importance regarding the likelihood of TRS.
3. What do you think is Kelly risk of developing TRS; mild moderate, high.
4. What are your reasons for placing Kelly in the risk category you assigned? (For each participant we will ask him/her for the reasons for the risk category).

**B**. We are particularly interested in determining a threshold for the risk prediction that clinicians are likely to use in clinical practice i.e. a threshold of the risk above which a clinician would consider using treatments such as Clozapine instead of first generation antipsychotics.

In a **hypothetical** situation as that depicted in Kelly case, if the model accurately predicts the likelihood of Jason developing TRS with a 70% accuracy, will you:

1. Consider using a treatment such as Clozapine, instead of a first line antipsychotics
2. Would the use of a decision treatment tool like this change your practice at all?
3. What in your view would be the best threshold at which you would consider using an alternative treatment strategy such as clozapine?

#

# **Appendix 6**

## **SPIRIT qualitative findings**

**Methods**

Qualitative data were generated through focus groups and semi-structured one-to-one interviews with clinicians (psychiatrists, pharmacists) responsible for managing the care of people with TRS.

**Sampling and recruitment**

Participants were purposively sampled to represent a broad range of clinical settings (acute inpatient, rehabilitation, liaison psychiatry, early intervention, and general adult psychiatry) from two NHS Trusts in England. Study information was shared over email by service leads at participating NHS Trusts. Services that participants were employed by received reimbursement to cover the cost of staff time to take part in data collection (based on typical hourly rate of pay).

**Data collection**

Focus groups were intended to last for between 60-90 minutes; interviews up to 60 minutes. A topic guide and fictitious case example were used to prompt discussion of challenges and opportunities for designing and embedding a clinical decision tool for TRS, based on the prediction model. The patient advisory group informed development of the topic guide and case examples. Data collection was conducted by TK, an experienced post-doctoral qualitative researcher, and supported by GA. All data collection took place remotely over MS Teams due to geographical distance and Covid-19 public health restrictions.

**Analysis**

Qualitative data were recorded, transcribed, and analysed using a thematic approach. TK led qualitative analysis and discussed preliminary findings with research team members to inform interpretation of data. Transcripts from focus groups and interviews were analysed together; comparisons were drawn between participants from within focus groups and interviews to support development of codes and identification of common themes. Topic guide questions were iteratively updated to support exploration of themes, as they developed. The concept of information power was operationalised to guide sampling, data collection and analysis.

**Findings**

Eleven clinicians (6 psychiatrists; 5 pharmacists) participated in two focus groups and three interviews, online via MS Teams. Participants were purposively sampled to represent a broad range of clinical settings (acute inpatient, rehabilitation, liaison psychiatry, early intervention and general adult psychiatry) from two NHS Trusts in England. Six participants were female (5 male) with an average age of 47 years (range: 24-64yrs) and average length of time in practice of 20 years (range: 8mths-40yrs).

Three themes are summarised below: multi-level challenges of identifying and managing TRS, benefits and barriers of/to the prediction model and clinical decision aid design preferences. Supporting data that has been de-identified is provided below to evidence data interpretation.

***Multi-level challenges of identifying and managing TRS***

Common challenges were identified at patient- (social stigma, treatment burden, poor compliance), clinician- (case complexity, accurate history taking, administrative burden of treatment, making the case for specific treatment) and service and policy-level (service capacity, service availability, treatment guidelines). These challenges established the context for discussion of the prediction model.

***Benefits and barriers of/to the prediction model***

All participants recognised the prediction model as a relevant resource with the potential to inform clinical decision-making and case discussion. All members of a multi-disciplinary team (i.e. psychiatrists, pharmacists, nurses, care coordinators) were considered likely to benefit from access to the prediction model. However, clinical challenges were identified as potential barriers to implementation and performance of the prediction model, such as, the challenge of recording an accurate patient history and confirming a diagnosis of schizophrenia. Participants acknowledged the importance of using the risk score in conjunction with other information to inform treatment decisions (e.g. history of relapse, hospitalisations, social factors). Participants maintained that treatment guidelines for TRS needed to be observed (i.e. non-response to two APs) and that a high risk score should not be perceived as approval to prescribe APs, such as, clozapine off-label. There was a difference in opinion about whether risk scores for TRS should be shared with patients; concerns were raised about the score potentially posing a barrier to communication, contributing to stigma and effecting attitudes to treatment.

***Clinical decision aid design preferences***

Participants identified opportunities to inform design of a clinical decision aid and support implementation. Access to published evidence reporting the development and validation of the prediction model was expected by participants to demonstrate robustness. Participants also requested a package of training to guide appropriate use, inform interpretation of risk scores and enable objective categorisation of risk – particularly as the list of predictors were commonly presented in practice. Participants described preferring an online or mobile phone applications format with linkage to the existing patient record management system a key factor when embedding in practice.

**Qualitative data to support thematic analysis.**

| **Theme** | **Supporting data** |
| --- | --- |
| **Multi-level challenges of identifying and managing TRS** | **Patient-level challenges**  *Stigma*  “We use terms like treatment resistant schizophrenia. When I talk to patients and especially families they say, ‘Oh gosh, it’s so dreadful because nothing is going to work’, you say, ‘Oh no, but we’ve got clozapine which does work’ and they say, ‘But it’s called treatment resistant, so therefore nothing works.’ So everybody just gives up. There’s a real feeling of hopelessness which I’m sure doesn’t help the medication efficacy to be frank. So I think that’s really important.” [FG2, Pha05, Specialist pharmacy]  *Compliance* and *treatment burden*  “Maybe if it’s treatment resistant then we know it is treatment resistant that we’re dealing with and we always look at clozapine as an option where possible. Sometimes it’s not possible because the patient is not willing for blood tests or we know that compliance is a major issue, so we might decide otherwise.” [FG2, Psy02, Inpatient service]  **Clinician-level challenges**  *Treatment burden (for clinicians)*  “I’ll just add another barrier to starting clozapine is just the work that is involved in starting clozapine. You know, the registration, the physical exam, checking all the sort of comorbidities. I think that is a barrier as well for medical staff to start on clozapine.” [FG1, Pha04]  *Case complexity*  “Because of the diagnostic uncertainties at the beginning and also the co-existence of other interventions to address the person with psychotic symptoms, I think it takes me and my colleagues a lot longer to say ‘no’ we’re dealing here with a treatment resistance schizophrenia not just a psychotic episode.” [Psy01]  *Accurate history-taking*  “Just to add on from that, quite often when I look through a patient’s history I’ll be like, ‘Oh they were discharged from a ward a year ago on this medication, therefore my assumption is they responded to that medication.’ Sometimes they really didn’t. They might’ve shown some marginal response, some partial response, but as soon as they were discharged in community they weren’t well. Or maybe they went AWOL and that’s actually why they were discharged and they were never treated. So sometimes sort of the management and the care isn’t an accurate reflection initially on the surface of what happened and what you think. So just because a patient was discharged from services doesn’t mean they were well at the time, which is often the assumption made. And there can be quite a lot of instability in their history and their accuracy and their accuracy and the time I’ll have the patient. So it can be quite hard to tame.” [FG1, Pha03]  **Service and policy-level challenges**  *Capacity*  “I think the other thing that we need to consider even to be pragmatic is really service provision, so how many patients people have in terms of I don’t know EI [Early Intervention] teams well, I do know actually because I used to work in one, but usually the EI teams have a high case load and the care coordinators have more than 15 patients so that means they cannot be seen very much and if they cannot be seen very much then they don’t get switched fast enough to the second antipsychotic medication and then they don’t get switched fast enough to clozapine.” [Psy05]  *Guidelines*  “But once we know if it’s treatment resistant schizophrenia we follow the guidelines as well as we can and if possible stick to the guidelines, existing ones, look at the choice and compliance before we can say, ‘Let’s try something else’ or not.” [FG2, Psy02, Inpatient service] |
| **Benefits and barriers of/to the prediction model** | **Potential use**  *Objective evidence*  Psy03: “Yeah and being able to say to other clinicians, ‘Actually we’ve used this, this is our clinical thoughts’, but we’ve also used this to support our clinical decision. And then to share it with the patients so they can start advocating and saying you know, ‘Give me a chance’.”  Pha05: “That’s a good point, yeah. Because often in psychiatry we don’t have like good numbers to be able to say, ‘Look, we’ve measured this, therefore this is the treatment you should have.’ It’s not like with high blood pressure or something like that. So yeah, I think for patients that could be really powerful in saying actually, ‘This really is the treatment for you’.” [FG2]  *Guidance on risk categorisation*  Pha04: “I guess it depends where you stratify, I can’t remember how you classified it, mild, moderate or high. I mean I said moderate, I put about 50%. So I would say yeah, that kind of feels about right. But I guess yeah, it depends on where you sort of put your markers really.”  Pha02: “I mean if we say that about a third of patients will be treatment resistant, it means to me therefore that this patient probably would be high risk. Maybe he’s got a 43% chance. You’re not going to see somebody who has got a 100% chance [laughs]. I don’t know how you interpret your results, but yeah that’s my feeling.”  Pha03: “I feel like 43% sort of accurately represents the information we have. But like >Pha04< and >Pha02<, I don’t know. I guess it’s all relative isn’t it? What are the majority of people scoring? What would I score? Kind of yeah, you’re not going to see 100%. I wouldn’t really expect to see much higher than 50% because I just think that’s already quite a hefty chance of developing it and you just don’t know.” [FG1]  **Clinical challenges**  “So many of our patients have them [predictors]. Like the comorbidity, the substance misuse is so high. The poor insight, it’s interesting how many have got a few of those factors already. Yeah, it’s quite worrying really.” [FG1, Pha04]  “I think also the diagnosis of schizophrenia is not always easy in that it’s the substance misuse that’s difficult. So is this a drug induced psychosis or is this a psychotic illness? And you know, quite often that’s quite hard, especially if it’s a first episode. Like with this guy he’s obviously got cannabis etcetera. Obviously there’s other things going on, but it’s not always I think straightforward to say schizophrenia or drug induced psychosis. I think that would be quite tricky.” [FG1, Pha04]  **Sharing risk score with patient**  “I don’t think that this score would help patients. I think quite often they’re either on board with clozapine or they’re not and as a clinical team we make that decision. And we have information out there for patients about clozapine. I think if you showed them a list of your criteria they would either agree or argue against it. I don’t know.” [FG1, Pha03] |
| **Clinical decision aid design preferences** | **Robust evidence**  “I would need to be sent studies, I would need literature and numbers in the context of literature… to make sure that it is a robust measure.” [Psy05]  **Training package**  “I think either. You know, I don’t mind either. I think what would be really useful is you know, once you have got the risk score right there needs to be an education package around it. What do the results mean in real life? What does the result mean for my patient? If they’ve got a 20%, 40%, whatever the percentage is, how does that translate for my patient?” [FG1, Pha02]  **Format preferences**  “I like apps and personally if I can do it on my phone and type it all in, tick boxes, lovely. I think a lot of people maybe a bit more old school would like the website. I think it’d be helpful to send the website links to healthcare professionals. I think you’d also need sort of a research paper or something behind it to give it some validation. But yeah, a website and an app.” [FG1, Pha03]  “Apps are more common than printed materials. An app would be really interesting. It would be good if it didn’t have a doctor-only connotation. If it was addressed mainly to care coordinators as that is where first discussions are likely to happen… and with a positive message that there are treatments to offer.” [Psy01] |

# **Appendix 7**

## **Description of predictor variables**

| **Variable** | **Definition** | **Reference(s)** |
| --- | --- | --- |
| Age | Age at first contact was defined as the age at  which a patient came into contact with mental health services for the first time following onset of psychotic symptoms.. | Fearon P, Kirkbride JB, Morgan C, et al. Incidence of schizophrenia and other psychoses in ethnic minority groups: results from the MRC AESOP study. Psychol Med 2006;36:1541–50  Ajnakina O, Lally J, Di Forti M, et al. Patterns of illness and care over the 5 years following onset of psychosis in different ethnic groups; the GAP-5 study. Soc Psychiatry Psychiatr Epidemiol 2017;52:1101–11 |
| DUP | Duration of untreated psychosis (DUP) was defined as the time between the date of onset of first psychotic symptoms to the date of treatment with antipsychotic medications. | Ajnakina O, Lally J, Di Forti M, et al. Patterns of illness and care over the 5 years following onset of psychosis in different ethnic groups; the GAP-5 study. Soc Psychiatry Psychiatr Epidemiol 2017;52:1101–11  Demjaha A, Lappin JM, Stahl D, et al. Antipsychotic treatment resistance in first-episode psychosis: prevalence, subtypes and predictors. Psychol Med. 2017 Aug;47(11):1981-1989. doi: 10.1017/S0033291717000435 |
| Drug use (yes/no) | Drug abuse/dependence was measured using a life chart. Participants were asked to rate their illicit drug taking and/or abuse over their life course. This was measured via a 7-point scale:  0 = none  1 = sporadic or occasional abuses reported, no evidence for frequent or regular use (i.e. less than one month)  2 = sporadic or occasional abuses reported, but there is reason to suspect frequent or regular use (i.e. more than one month)  3 = frequent or regular use definitely present (i.e. more than one month)  4 = substance abuse (maladaptive use leading to any of the following: (i) failure to fulfil major role obligations due to substance, (ii) substance exacerbating or leading to social or interpersonal problems, (iii) recurrent abuse when physically hazardous (i.e. driving) or substance related legal problems  5 = substance dependence (maladaptive use leading to three of the following: (i) increased tolerance, (ii) symptoms of withdrawal, (iii) substance taken in larger amounts over a longer period than originally intended, (iv) persistent desire or unsuccessful attempts to cut down, (v) much time spent in activities to obtain the substance or recovering from effects, (vi) impairment of social, occupational or recreational activities due to substance, (vii) persistent use despite harmful physical or psychological effects,  7 = drug taking a definite possibility but impossible to assess the frequency and extent of use.  If participants were identified as substance abuse or substance dependence, they were asked to specify time periods. Participants were also asked to specify the nature of the substance(s) taken and age of first use. For each drug, a score of 0 = no, 1 = yes, 2 = suspected/uncertain, 8 = not applicable, 9 = no information/impossible to access. | Reininghaus U, Dutta R, Dazzan P, et al. Mortality in schizophrenia and other psychoses: a 10-year follow-up of the ӔSOP first-episode cohort. Schizophr Bull. 2015 May;41(3):664-73. doi: 10.1093/schbul/sbu138.  Morgan C, Dazzan P, Morgan K, Jones P, Harrison G, Leff J, Murray R, Fearon P; AESOP study group. First episode psychosis and ethnicity: initial findings from the AESOP study. World Psychiatry. 2006 Feb;5(1):40-6. PMID: 16757995; PMCID: PMC1472260. |
| Alcohol use (yes/no) | Alcohol abuse/dependence was measured using a life chart.  Participants were asked about their drinking habits over the life course. The scoring was as follows:  0 = does not drink at all  1 = only occasional social drinking (10 units or less per week)  2 = moderate alcohol use (21 units or less per week)  3 = excessive alcohol use (more than 21 units per week regularly)  4 = alcohol abuse (maladaptive use leading to any of the following: (i) failure to fulfil major role obligations due to alcohol, (ii) substance exacerbating or leading to social or interpersonal problems, (iii) recurrent abuse when physically hazardous or alcohol related legal problems  5 = alcohol dependence (maladaptive use leading to three of the following: (i) increased tolerance, (ii) symptoms of withdrawal, (iii) alcohol taken in larger amounts over a longer period than originally intended, (iv) persistent desire or unsuccessful attempts to cut down, (v) much time spent drinking or recovering from the effects, (vi) impairment of social, occupational or recreational activities due to alcohol, (vii) persistent use despite harmful physical or psychological effects  9 = no information/not known.  If rated a score of 3,4 or 5, participants were asked more about the nature of their alcohol intake. If rated a score of 4 or 5, participants were asked about date periods of alcohol abuse or dependence.  In the GAP study, alcohol use was measured in terms of units consumed per week:  >0-14 units a week  >15<28 units a week  >29 units a week  None  Recreational drugs were measured with an initial YES/NO answer, and participants who stated yes were asked to provide details of:  Substance name  Age when started/stopped  How long used for | Reininghaus U, Dutta R, Dazzan P, et al. Mortality in schizophrenia and other psychoses: a 10-year follow-up of the ӔSOP first-episode cohort. Schizophr Bull. 2015 May;41(3):664-73. doi: 10.1093/schbul/sbu138.  Morgan C, Dazzan P, Morgan K, Jones P, Harrison G, Leff J, Murray R, Fearon P; AESOP study group. First episode psychosis and ethnicity: initial findings from the AESOP study. World Psychiatry. 2006 Feb;5(1):40-6. PMID: 16757995; PMCID: PMC1472260. |
| Premorbid adjustment (NART) | The National Adult Reading Test full-scale IQ (NART-FSIQ) was used to measure premorbid adjustment. This scale produces reliable, standardised estimates of the Wechsler Adult Intelligence Scale (WAIS-IV) IQ test and is a widely adopted method for estimating premorbid intelligence. | Bright P, Hale E, Gooch VJ, et al. The National Adult Reading Test: restandardisation against the Wechsler Adult Intelligence Scale – fourth edition. Neuropsychological Rehabilitation 2018; 28(6):1019–1027 |
| Diagnosis of schizophrenia or schizoaffective disorder (instead of psychotic depression or psychotic mania) | The diagnosis was based on participants meeting the International Classification of Diseases (ICD)-10 criteria for a diagnosis of non-affective (F20–F29) or affective (F30–F33) psychosis, validated by administration of the Schedules for Clinical Assessment in Neuropsychiatry (SCAN). | Murray RM, Mondelli V, Stilo SA, et al. The influence of risk factors on the onset and outcome of psychosis: what we learned from the gap study. Schizophr Res 2020;225:63–8  Ajnakina O, Lally J, Di Forti M, et al. Patterns of illness and care over the 5 years following onset of psychosis in different ethnic groups; the GAP-5 study. Soc Psychiatry Psychiatr Epidemiol 2017;52:1101–11 |
| Family history of psychosis | The Family Interview for Genetic Studies (FIGS; <https://www.nimhgenetics.org/interviews/figs>) was used to obtain information about the participant’s family history of mental health problems. To maximise genetic risk, only information on first-degree relatives (participant’s biological mother and father, full siblings and children) was used. The FIGS consensus diagnoses were divided into several familial risk variables. First, a ‘family mental illness’ variable referred to the presence (1) or absence (0) of current or past psychosis, mania or depression in at least one first-degree relative. A ‘family psychosis’ variable denoted the presence (1) or absence (0) of a current or previous diagnosis of psychosis in at least one first-degree relative. A ‘parental mental illness’ variable was also created that indicated the presence (1) or absence (0) of a current or previous diagnosis of psychosis, mania or depression in at least one biological parent. Similarly, a variable for ‘parental psychosis’ was created that denoted the presence (1) or absence (0) of current or past psychosis in at least one biological parent. | Trotta A, Di Forti M, Iyegbe C, et al. Familial risk and childhood adversity interplay in the onset of psychosis. BJPsych Open 2014 1:6-13. doi: 10.1192/bjpo.bp.115.000158 |

# **Appendix 8**

## **All calibration plots**

# **Appendix 9**

## **All decision curve analysis plots**

# **Appendix 10**

## **SPIRIT protocol paper**
